# Measuring brand association strength with EEG: A single-trial N400 ERP study

**DOI:** 10.1371/journal.pone.0217125

**Published:** 2019-06-10

**Authors:** Flavio Camarrone, Marc M. Van Hulle

**Affiliations:** Laboratory for Neuro- & Psychophysiology, Department of Neurosciences, KU Leuven—University of Leuven, Leuven, Belgium; University of Pennsylvania, UNITED STATES

## Abstract

Companies need to ensure that customers perceive their brands as intended, with strong and unique associations, when facing a competitive market. Traditionally, brand associations are measured using conventional techniques such as surveys and questionnaires albeit both conscious and unconscious factors can influence the collected data and the outcome of a campaign. Neuromarketing can shed light on how the customer’s brain processes marketing stimuli. We report here on an EEG study aimed at gauging mental associations with brands. We focus on the N400 event-related potential, an EEG component most strongly elicited in response to a concept unrelated to a preceding concept. We considered two video on demand brands, *Netflix* and *Rex&Rio*, and selected a set of words grouped in 4 categories that were either related (*Television*, *Relaxation*, and *Price*), in varying degrees, or unrelated (*Unrelated*) to the said brands. The experiment started with both brands’ TV commercials, as a common reference for our participants. We then applied a semantic priming paradigm in which a brand logo (“prime”) was followed by a word (“target”), and the strength of the N400 response to the word used as an inverted measure of the association strength with the brand logo. We clustered N400 responses to identify, for each brand, natural groups of associated words. As a result, for *Netflix* the cluster with the smallest N400 responses (i.e., strongest associations) consisted of words related to *Television* but for *Rex&Rio* it consisted of words related to *Relaxation*. We also evaluated the relationship between the two brands and determined which associations they share or which ones not. It turned out that associations related to *Relaxation* and *Television* distinguish the two brands. Interestingly, survey data did not show any difference between the two brands as they were equally associated with *Television* and *Relaxation*. These findings show that our N400 technique can reveal brand associations, and natural categories thereof, that would otherwise go unnoticed when using conventional surveys.

## Introduction

A strong and differentiated brand is a key ingredient in securing a company’s performance in the market. The concept brand has been defined as “a speech flowing from the sender to a receiver” [1]. The marketer’s main challenge is to align the company’s desired portraying of the brand (brand identity) with what is actually perceived by the customer (brand image) [2],[3]. This question also led to an increasing number of studies on how a customer perceives marketing stimuli [4]-[7]. Some authors believe this depends on brand memories and how they relate to brand associations [8]-[10]. The importance of brand associations is also stressed by O’Cass and Lim [11] who found that consumers use such associations to differentiate brands. In addition, many studies such as [12]-[15] show that influencing brand associations can have an impact on brand equity (i.e., the commercial value of the brand) and the customer’s future brand buying behavior. In other words, brands with strong and unique associations are more competitive [16] and perform better in securing financial returns [17].

Several techniques have been proposed to measure the effectiveness of brand associations [18]-[20]. Traditionally, marketers resort to questionnaires, focus groups and in-depth interviews [21]-[25]. One example is Krishnan’s study [15] where the consumer’s perception of a brand was gauged in terms of awareness associations using a traditional survey. In line with the theory of spreading activation [26],[27], he described brands as clusters of associations organized in a (semantic) network with nodes representing brands, products, or attributes linked to other nodes by their degree of association (e.g., the node *Nike* is linked to the node *athletic shoes*). After identifying the semantic network, the value of a brand can be calculated and, eventually, differences between mature and new brands examined. The results show that the memory network model is a valid tool for brand evaluation. Although still popular in marketing, interviews have been criticized for influencing the respondent by the line of questioning [28] and questionnaires for falling short in revealing the respondent’s true preferences [29],[30].

**Fig 1:**
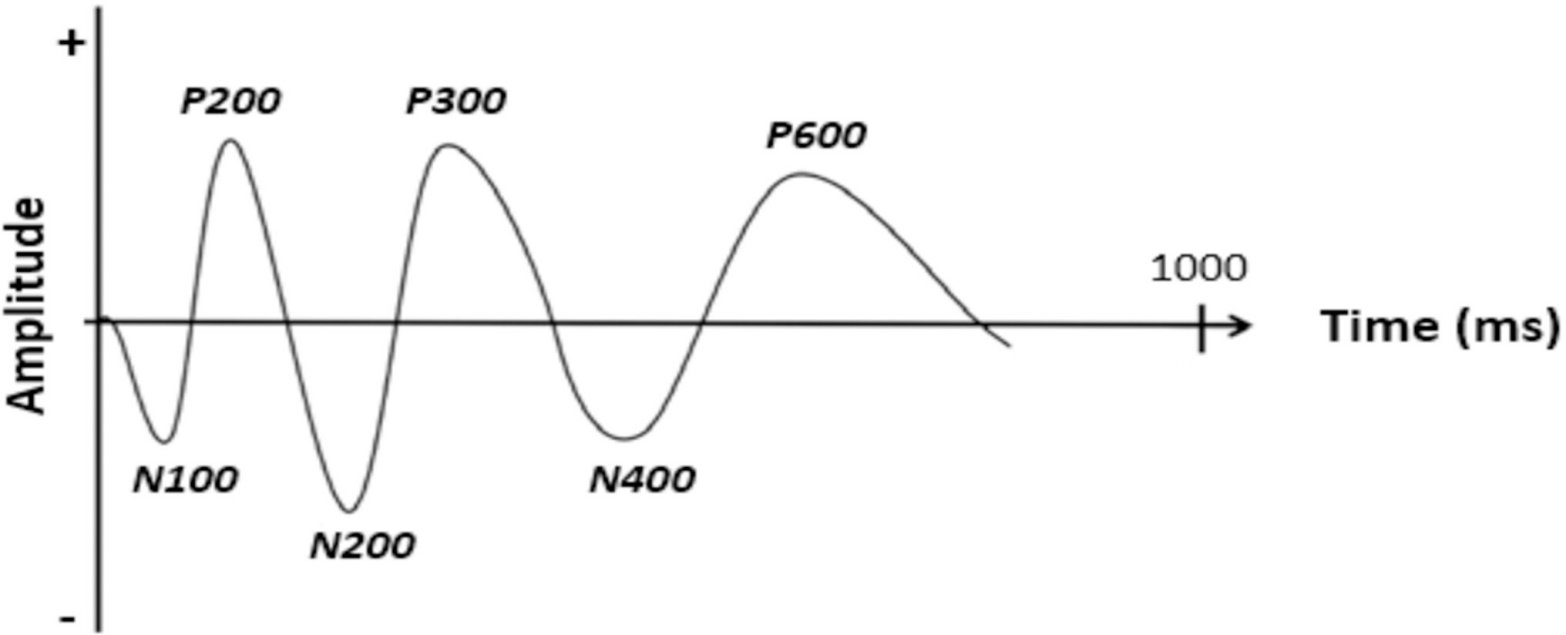
Concept plot of an Event-related potential (ERP) evoked in response to a stimulus. An ERP consist of a series of characteristic amplitude deflections following stimulus onset, called ERP components, that are referred to by a letter (N/P), to indicate their polarity (negative/positive), followed by a number to indicate their latency in milliseconds (e.g., P300 as it occurs around 300 milliseconds) or their position in the sequence (N1, P2, etc., not shown) [38].

Brain imaging tools, routinely used in clinical examinations and neuroscience research, have been adopted in Neuromarketing to collect more veridical customer responses, as brain activity is to a large extent unbidden. Equipped with such tools, the (neuro)marketer can probe the mental processes related to consumer behavior, a valuable asset compared to what is offered by traditional marketing techniques [8],[31],[32]. Electroencephalography (EEG) has become particularly popular for its excellent temporal resolution and its ability to collect and track brain activity in a non-intrusive way. In addition, EEG devices have become relatively inexpensive, portable and easy to set-up. Prior to EEG recording, electrodes are placed on the subject’s scalp, after which voltage fluctuations are recorded that reflect coordinated neural activity. An EEG pattern that recently gained interest in Neuromarketing is the event-related potential (ERP), a characteristic sequence of positive and negative deflections in EEG amplitude, time-locked to the stimulus of interest (Fig 1). ERPs are divided into 2 categories: the early amplitude components (below 200 milliseconds) that relate to the physical appearance of the stimulus (sensorial components), and the later amplitude components that are modulated by stimulus involvement, meaning, memory, recall, etc. (cognitive components).

Several recent neuromarketing studies have explored the relation between certain ERP components and brand stimuli [33]-[36]. In a recent study, Nedelko et al. [37] focused on the N400 component for testing how brand statements were perceived in participants’ minds. In their experiment, the authors used sentences in which congruent and incongruent brand associations were presented before brand names (e.g. “for me jokes and Nivea are associated”), and evaluated the N400 amplitude in response to the presentation of the brand name. The outcome of this study showed that anomalous brand statements evoked larger N400 responses.

The aforementioned findings support the theory that brands are represented in our minds as sets of associations that are activated when the brand names are shown. EEG-ERP experiments can help marketers to understand how the brand is perceived, e.g., when the participant perceives the suggested link between brand name and targeted attributes as incongruent, then his/her N400 responses will be larger.

In this study, we take the application of the N400 in Neuromarketing one step further. Our goal is to propose a new technique based on N400 responses for assessing mental associations with a specific brand (brand image) and to evaluate how these differ from the intended associations (brand identity). To this end, we first showed TV commercials of two video on demand brands as a common reference for all participants. Next, we applied a semantic priming paradigm in which pairs of brand logos (“primes”) and words (“targets”) were briefly flashed in succession and the N400 responses to the word stimuli extracted for measuring the association strength with the brand logos. In order to extract the N400 response to a single target word, we propose a modified version of the spatiotemporal beamformer algorithm (see further stLCMV). The N400 responses to each brand were then subject to a cluster analysis and the found clusters compared with the 3 word categories. Finally, in order to unveil the brand associations shared or not shared between the brands, clusters were intersected.

## Materials and methods

**Fig 2:**
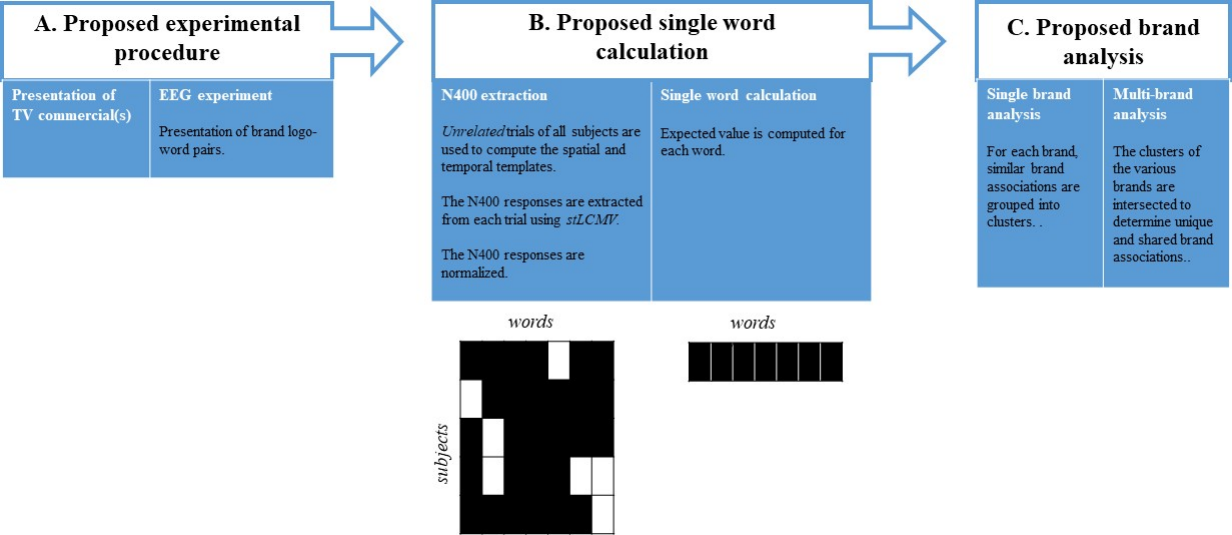
Diagram summarizing our proposed method.

In this study we propose a three-step method to measure brand associations based on the N400 ERP component: (A) a classic priming experiment in which participants view images of brand logo’s followed by words while recording their EEGs; (B) the processing of the EEG recordings, the extraction of the ERPs, and the quantification of the brand logo–word association strengths in terms of N400 ERP component strengths; (C) a clustering analysis of the N400 results to identify groups of words that are strongly associated to the brands and individual words that are shared (common associations) or not shared (distinctive associations) by the brands. A graphical representation of the proposed method is summarized in Fig 2.

### Participants

Twenty-six native Flemish-Dutch speaking university students (14 female and 12 male) with age between 18 and 28 years (mean age 22 ±2.5) participated in the main experiment that included EEG recording (see further). All participants had normal or corrected to normal vision and no history of neurological or psychiatric abnormalities. Ethical approval for this study has been granted by an independent ethical committee (“Commissie voor Medische Ethiek” of UZ Leuven) and all subjects signed the informed consent form prior to the experiment and after being informed by its purpose and design.

### Brands chosen

For our study we selected 2 brands, *Netflix* and *Rex&Rio*, companies operating in Belgium but with different degrees of market penetration. *Netflix* is a so-called video on demand service that streams movies and series. *Netflix* is active in Belgium since September 19, 2014. *Rex&Rio* is owned by *Telenet*, which is mostly known as an internet provider but also offers services such as high definition (HD) television, fixed and mobile telephony, and high-speed broadband internet. On September 23, 2014, *Telenet* launched *Rex&Rio* whereby *Rex* and *Rio* refer to two former cinemas in *Telenet*’s hometown (Mechelen, Belgium). The service *Rex* consists of a library of Flemish television series and movies, shows, children programs and documentaries whereas *Rex&Rio* additionally provides international series and movies, mostly prestigious series from HBO. *Netflix* and *Rex&Rio* are both available on payable subscription only. The most striking difference is their price: *Netflix* costs € 8 per month, and *Rex&Rio* € 24.94 per month (prices as reported in October 2014). Shortly after performing our experiment, *Rex&Rio* was incorporated into a new service called *Play* and *Play More* (announced in December 2014).

### Word association selection

In accordance with Heylen’s definition of brand identity–"the explicit, external features that are observed by the rational" [39]–, we first wanted to chart the brand identity categories using an on-line survey to which 28 participants responded (different from the ones that participated in the main experiment). Participants were divided in 2 equal groups and each group was asked to evaluate either *Netflix* or *Rex&Rio* in a three-stage process: the participant was asked to (1) watch the TV commercial of the brand, then (2) write down the first 5 associations that came to mind (free association task) and, finally, (3) provide association scores (from 1 to 10) to 20 words that are generally associated with a video on demand service. The outcome of the free association task is shown in Table 1 where for each association we report the percentage of responses (Rating), whereas in Table 2 we indicate the score as an average over all participants.

**Table 1:** Free association task. Summary of 5 associations returned by the 14 Dutch-speaking respondents per brand group, after viewing the brand’s TV commercial. Note that the responses were in Dutch; English translations are provided for illustrative purposes. The results are ranked according to the percentage of total associations vs. number of participants. For brevity, only the top 10% words are shown.

*Rex&Rio* (Group 1)			*Netflix* (Group 2)		
Word		Rating (%)	Word		Rating (%)
Dutch	English		Dutch	English	
Film	*Movie*	93	Series	*Series*	71
Kinderen	*Children*	71	Film	*Movie*	64
Cinema	*Theater*	50	Handig	*Handy*	43
Televisie	*Television*	43	Schermen	*Displays*	21
Series	*Series*	36	Smartphone	*Smartphone*	21
Telenet	*Telenet*	36	Bekend	*Familiar*	21
Goedkoop	*Cheap*	21	Televisie	*Television*	14
Familie	*Family*	14	Goedkoop	*Cheap*	14
Goesting	*Desire*	14			

**Table 2:** Association rating task. Summary of scoring a list of 20 words, scores between 1 and 10, depending on their relevance for the TV commercials. For each word, the final score is calculated as the mean of all participants’ scores.

Word		Score	
Dutch	English	*Rex&Rio* (Group 1)	*Netflix* (Group 2)
Ontspanning	*Relaxation*	8.15	8.86
Abonnement	*Abonnement*	8.31	8.71
Plezier	*Pleasure*	7.61	7.79
Televisie	*Television*	9.23	9.14
Makkelijk	*Easy*	4.69	8.21
Goedkoop	*Cheap*	3.54	6.5
Programma	*Program*	5.92	5.79
Thuis	*Home*	6.62	6.29
Vrienden	*Friends*	7.08	4
Film	*Movie*	9.46	9.07
Flexibel	*Flexible*	5.69	8.07
Nuttig	*Useful*	3.31	5.14
Bioscoop	*Theater*	5.77	3.21
Handig	*Handy*	5.31	7.43
Series	*Series*	6.62	9.64
Mobiel	*Mobile*	4.15	6.29
Documentaire	*Documentary*	4.08	4.07
Jeugdig	*Youthful*	7.08	5.5
Sport	*Sport*	3.08	2.07
Noodzakelijk	*Necessary*	1.92	1.71

We retained the ten most frequent words returned words in the free association task, and those with the highest association ratings for each of the 3 categories (*Price*, *Relaxation*, *Television*), supplemented by 10 unrelated words, unrelated to the brand categories (*Unrelated* category). The final word list is shown in Table 3.

**Table 3:** List of 40 Dutch words used in the EEG experiment with their English translations (for illustration purposes only).

Television		Relaxation		Price		Unrelated	
Dutch	English	Dutch	English	Dutch	English	Dutch	English
Televisie	*Television*	Ontspanning	*Recreation*	Duur	*Expensive*	Banaan	*Banana*
Film	*Movie*	Rust	*Rest*	Geld	*Money*	Vlieg	*Fly*
Serie	*Series*	Vrienden	*Friends*	Tijd	*Time*	Drinken	*Drinking*
Aflevering	*Episode*	Plezier	*Fun*	Rijk	*Rich*	Mexico	*Mexico*
Tekenfilm	*Cartoon*	Genieten	*Enjoy*	Luxe	*Luxury*	Lamp	*Lamp*
Kijken	*Watching*	Tof	*Cool*	Prijs	*Price*	Gang	*Hallway*
Kinderfilm	*Child movie*	Lachen	*Laugh*	Euro	*Euro*	Raam	*Window*
Bioscoop	*Theater*	Relaxen	*Relax*	Betalen	*Pay*	Auto	*Car*
Thuis	*At home*	Rustig	*Quiet*	Kosten	*Expense*	Fabriek	*Factory*
Nieuws	*News*	Kalm	*Calm*	Goedkoop	*Cheap*	Lactose	*Lactose*

### N400 ERP

When conducting an experiment with sentences, Kutas and Hillyard [40] noticed that a negative EEG component called N400 ERP, with range 250-500ms and peaking around 400ms, was elicited in response to incongruent sentence final words. For example, when words were displayed in rapid sequence and the last word of the sentence is out of context, e.g., I drink coffee with milk and socks, then an N400 is evoked when the word *socks* is displayed. Kutas and Hillyard [41] demonstrated that the N400 is also evoked in response to a word such as *sock* (target) when preceded by an unrelated word such as *dog* (prime), but absent in response to a related word such as *cat* (target). The N400 amplitude has been shown to inversely relate to the association strength between prime and target [42],[43],[44]. The basis of semantic priming is the *theory of spreading activation* [26],[27] according to which a presented word pre-activates related words recalled from memory, hence, when one of those is presented next, its processing is facilitated. The priming effect is thus seen as evidence that our semantic system is closely coupled to the memory system. The N400 ERP can be elicited consciously or even subconsciously (masked priming [45]) in response to meaningful stimuli presented out of context or in an unrelated context (for review, see [46],[47]).

### Main experiment

Participants to the main experiment were tested while sitting in front of an LCD screen. The distance from the screen was around 1m. The whole experiment consisted of four parts: (1) presentation of TV commercials of the 2 brands, (2) eye movement calibration, (3) subject training, and (4) core EEG experiment.

**Fig 3:**
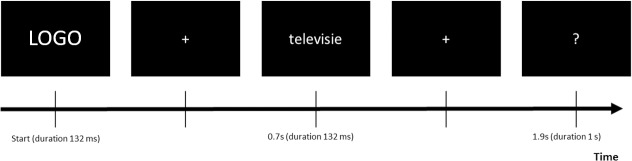
Example of prime and target stimulus presentation. The sequence is presented as prime (brand logo), crosshair, target stimulus, crosshair, and question mark whereby the latter prompts the participant for a button press response.

For each brand, a TV commercial was displayed that served as a common reference for our participants. The core of the experiment relies on a classic priming paradigm: two stimuli, called prime and target, are presented in rapid succession (see further for timing details). The prime was the brand logo of either *Rex&Rio* or *Netflix* and the target a Dutch word taken from the 40 listed in Table 3. Each target was shown twice during each session, once per brand, and randomly paired with each brand’s logo. In total, the experiment consisted of 80 prime-target pairs (2 brands × 4 categories × 10 words) presented in two blocks of 40 prime-target pairs, separated by a 10-minutes break. Prime and target stimuli were presented in the center of the screen. For the target stimuli white letters on a black background were used. The subjects saw the brand logo for 132 ms and the target word also for 132 ms, with a 700 ms interval between the two (inter-stimulus interval). After showing the target word, a crosshair appeared to keep the subject focused and, after a delay of 1 s, a question mark during 500 ms after which participants had to indicate whether they thought the pairs were associated or not (“yes”/“no”), by pressing the corresponding mouse buttons, or when they were uncertain of any association, not to press any button (“no answer”). The button press task was introduced to keep our participants engaged in the experiment. Our participants had 500 ms to press the button, otherwise the trial was marked as “no answer”. Note that the button press occurred outside the expected time range of the N400 component so as not to contaminate the latter with button press-related ERPs [48]. The responding hand, as well as the mapping of the button to the “yes”/“no” answer were counterbalanced, across participants. A scheme of the stimulation is shown in Fig 3. Prior to the experiment, our participants were trained on a prime-target paradigm using word pairs unrelated to the core EEG experiment (thus, also no logos). All stimuli were displayed using Matlab’s Psych-toolbox [49].

### Electroencephalography recording

Participants were tested in a sound-attenuated, darkened room with a constant temperature of 20 degrees, sitting in front of an LCD screen. EEG was recorded continuously using 32 active electrodes, evenly distributed over the entire scalp (positioning and naming convention following a subset of the extended 10–20 system), with a BioSemi ActiveTwo system (BioSemi, Amsterdam, the Netherlands), operating at a sampling rate of 2048 Hz, and an electro-oculogram (EOG) using the set-up of [50]. In addition, to re-reference BioSemi’s common mode sense reference [51] (CMS, positioned next to electrode Pz) to a mastoid reference, to better detect the N400 component, two additional electrodes were placed on both mastoids and their EEG signals averaged. The total duration of the experiment, excluding electrode setup, was about 20 minutes.

### Data preprocessing

The EEG signal was re-referenced offline to a mastoid reference, then filtered using a 4th order Butterworth filter with range 0.5–30 Hz and the initial 2048 Hz sampling rate downed to 256 Hz (including anti-aliasing). The EOG signal was used to remove eye artifacts following the AAA method proposed in [50]. Trials with EEG amplitudes exceeding 70μV on any of the channels were considered to be affected by muscle artifacts and were discarded, as well as trials lacking button press responses (i.e., “no answer”). EEG epochs were extracted from -100 to 1000 ms to the onset of the stimulus, with the 100ms pre-stimulus period used for baseline correction. Before applying the multivariate analysis methods, the signal was further downsampled to 80Hz to reduce dimensionality, as suggested in [43].

### Behavioral analysis

We labeled each trial with a 1 or 0 depending on the “yes” or “no” button press responses, respectively, and grouped trials into one of the prior-chosen categories *Television*, *Relaxation*, *Price*, and *Unrelated*. For the statistical analysis we performed pairwise comparisons among the categories using a mixed model, known to be robust to unbalanced data [52]. The behavioral response was used as a dependent variable with category label as independent variable. Subject was entered as random effect. P-values obtained with pairwise comparisons were corrected using the False Discovery Rate (FDR) method [53]. A significance level of p = 0.05 was used across the entire analysis.

### Proposed method

First, we measure single word associations using a 2-step method: N400 amplitude extraction and single word calculation. Then, we propose a novel method for within- and between-brands analysis: single-brand clustering and multi-brand comparison. Numerical analyses were performed in Python [54]. Both methods are further detailed.

#### Proposed single word calculation: N400 extraction

For each trial, we applied our recently developed spatiotemporal extension of the linearly constrained minimum variance beamformer (stLCMV) [43], which relies on the targeted ERP component’s temporal and spatial response profiles, further called templates. In contrast with [43], where 2 predefined categories were required (related and unrelated), in the current study, we constructed these templates using only the *Unrelated* category trials of all subjects, for both companies, so as not to make any prior assumptions about relatedness of the trials. The *Unrelated* trials were then removed from further N400 analysis. Subjects with less than 30 useful trials were excluded. For the remaining subjects, the N400 was extracted from single trials by applying the aforementioned stLCMV beamformer and its (scalar) output taken as N400 response strength. In addition, as recently proposed by Van Petten [55], for each subject individually, we converted the N400 responses into z-scores to overcome the variability in EEG amplitudes among subjects.

#### Proposed single word calculation: Single word calculation

Each target word is then configured as a vector with as many components as there are subjects. Since for some subjects trials had to be removed (see *Data preprocessing*), some words/vectors have missing components. A subject’s response to a target word can be regarded as an observation, which we assume to be independent and identically multivariate normal distributed, and a missing response to be missing at random (MAR) [56]. Given this assumption, we adopted the standard implementation of expectation conditional maximization (ECM) [57] as provided by the MATLAB Financial Toolbox. The main advantage of ECM is that it can also estimate the mean when some observations are missing. As a result, we obtained the expected response of each target word, and whence a set of 30 (3 categories x 10 words) values, for each of the 2 brands.

#### Proposed brand analysis: Single-brand analysis

Albeit we initially collected target words belonging to 3 categories, we did not assume the existence of those categories in our N400 analysis: instead of considering pre-defined categories, we aimed to identify “natural” ones by adopting a data-driven clustering analysis of N400 responses. In this way, the found clusters represent natural categories of words according to their N400 responses with the most negative N400 ones representing the most unrelated (non-associated) words and vice-versa. We adopted an automatic approach to determine the optimal number of clusters (for the full procedure see S1 Appendix).

#### Proposed brand analysis: Multi-brand analysis

A further step in our multi-brand analysis is the intersection of the clusters found in the *Single-brand analysis* with the aim to list the target words that are associated with both brands (associations in common) or not (distinctive associations).

## Results

When inspecting the data from the 26 subjects (see *Data preprocessing*), 129 trials had “no answer”,199 an EEG amplitude exceeding 70μV, and 15 both. After removing 313 trials, a total of 1767 trials remained: 214 (*Price*), 215 (*Relaxation*), 227 (*Television*), 221 (*Unrelated*) for *Netflix*; 214 (*Price*), 226 (*Relaxation*), 225 (*Television*), 225 (*Unrelated*) for *Rex&Rio*. Then, we started evaluating the behavioral responses as described in the *Behavioral Analysis* section. The result is summarized in Table 4 (recall that related words are associated with 1, unrelated with 0). The analysis shows that the two companies had similar results. Note that the category *Unrelated* differs from the other categories, for both brands (p < .001 for all pairwise comparisons). Moreover, its mean is close to zero, confirming that subjects considered the majority of those words as unrelated to the brands. This allows us to assume that words from *Unrelated* elicit the strongest N400 responses. In addition, *Price* seems to be placed in between *Relaxation* and *Television* and *Unrelated*. Also, there is no difference between *Television* and *Relaxation*. The complete set of responses can be found in the S1 Table.

**Table 4:** Pairwise comparisons using a mixed model based on behavioral responses.

**A. Netflix**				
	*Relaxation*	*Price*	*Television*	*Unrelated*
*Relaxation*	-	p = 0.008*	p = 0.782	p < 0.001*
*Price*	p = 0.008*	-	p = 0.005*	p < 0.001*
*Television*	p = 0.782	p = 0.005*	-	p < 0.001*
*Unrelated*	p < 0.001*	p < 0.001*	p < 0.001*	-
Mean / Std	0.766 / 0.42	0.658 / 0.48	0.777 / 0.42	0.041 / 0.2
**B. Rex&Rio**				
	*Relaxation*	*Price*	*Television*	*Unrelated*
*Relaxation*	-	p < 0.001*	p = 0.637	p < 0.001*
*Price*	p < 0.001*	-	p < 0.001*	p < 0.001*
*Television*	p = 0.637	p < 0.001*	-	p < 0.001*
*Unrelated*	p < 0.001*	p < 0.001*	p < 0.001*	-
Mean / Std	0.77 / 0.42	0.595 / 0.49	0.789 / 0.41	0.072 / 0.26

P-values were corrected using False Discovery Rate (FDR). Mean and standard deviation (“Std”) for the different categories are also shown. Upper table: results associated with Netflix. Lower table: results associated with Rex&Rio. Note that the symbol “*” indicates the p-values smaller than 0.001.

**Fig 4:**
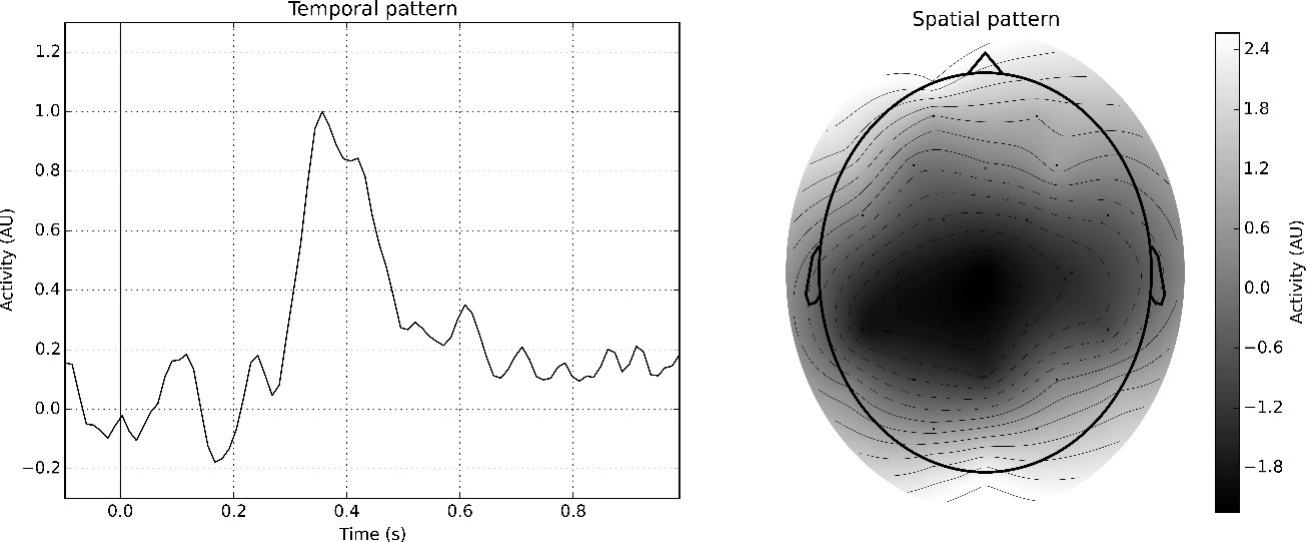
Beamformer templates. Beamformer templates obtained using the *Unrelated* category to extract N400 responses. The templates are in accordance with the N400 literature [43]: the temporal template shows a maximal amplitude around 400ms (left panel), whereas the spatial template shows that the latter occurs over the centro-parietal area (right panel).

We used the 446 trials (225 for *Rex&Rio*, 221 for *Netflix*) belonging to the *Unrelated* category to calculate the templates for the beamformer. The templates are shown in Fig 4. In accordance with the N400 literature, these templates reveal a maximal activation over centro-parietal electrodes (cf., spatial template in Fig 4), and a clear peak around 400 ms (cf., temporal template). After removing the *Unrelated* category trials, the beamformer is applied to the remaining trials, and the outputs noted as the corresponding N400 response strenghts. In total, 24 subjects and 1273 trials (628 for *Netflix* and 645 for *Rex&Rio*, respectively) were retained for further analysis (subjects with less than 30 trials were removed). The N400 response strengths were then converted into z-scores, for each subject individually, to account for inter-subject variability [56]. The outcome is shown in S1 Fig. Finally, the N400 response to each word is calculated in terms of the expected value obtained from all subjects’ responses (see *Single word calculation*).

We performed 3 different analyses: we first focused on *Netflix* (Analysis 1) and *Rex&Rio* (Analysis 2), and then performed a multi-brand analysis to identify the associations shared and not shared by the two brands (Analysis 3).

### Analysis 1: *Netflix*

**Fig 5:**
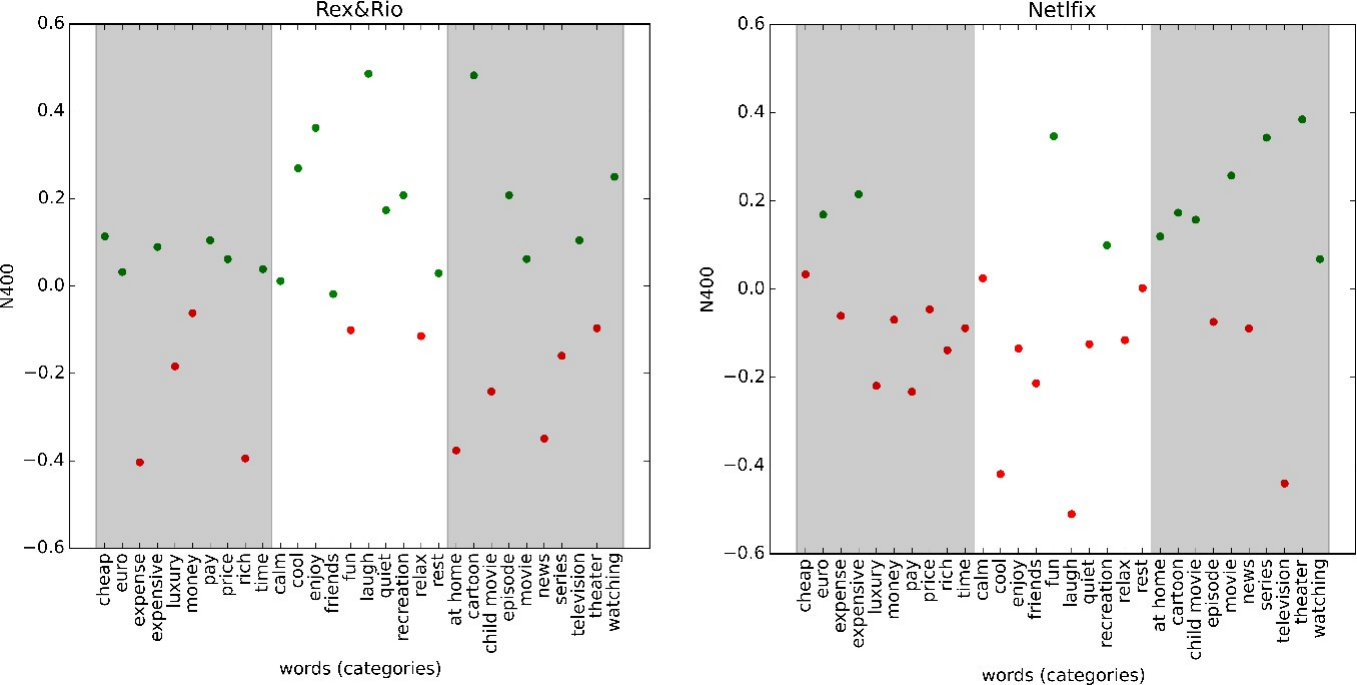
Clusters and categories representation. N400 responses vs. words, colored according to the clusters (green versus red, Cluster 1 versus Cluster 2) and organized according to category (from left to right, *Price*, *Relaxation*, *Television*). Left panel: *Rex&Rio* associations. Right panel: *Netflix* associations.

After performing the stability analysis (see S1 Appendix for details), we identified two optimal clusters: Cluster 1 (mean -0.15) consisting of 11 words and Cluster 2 (mean 0.21) of 29 words. These clusters represent the related (Cluster 1) and unrelated (Cluster 2) associations with *Netflix*. In order to statistically verify whether the N400 responses differed between clusters, we used a mixed model approach. For each subject, the single N400 response, labeled according to target word, was used as dependent variable and cluster label as independent variable. Subjects and target words were entered as random effects. Statistical analysis was performed in R using the lmer4 [52] package that includes the functions needed to implement the linear mixed model. We found a significant difference between the two clusters (F = 22.768, p < 0.001). Note also that the brand is mostly associated with *Television*, as Cluster 1 (most positive N400 responses) contains mostly words from this category (7 out of 11). Moreover, the brand is not related with *Relaxation* and *Price* as, for both categories, 8 words out of 10 are in Cluster 2 (most negative N400 responses) (Fig 5, right panel).

### Analysis 2: *Rex&Rio*

Similar to the *Netflix* case, also for *Rex&Rio* we found two optimal clusters: Cluster 1 (mean -0.23) consisting of 29 words and Cluster 2 (mean 0.16) of 11 words. Again, using a mixed model, we found that the two groups differed significantly (F = 20.426, p <0. 001). Again, Cluster 1 contains the most positive N400 response strengths, whereas Cluster 2 the most negative ones. From the distribution of the N400 responses, one observes that the brand tends to be associated with *Relaxation*, as 8 words out of 10 are in Cluster 1, and unrelated with *Price*, as the words from this category are mostly negative or close to zero (Fig 5, left panel).

### Analysis 3: *Netflix* and *Rex&Rio* comparison

**Fig 6:**
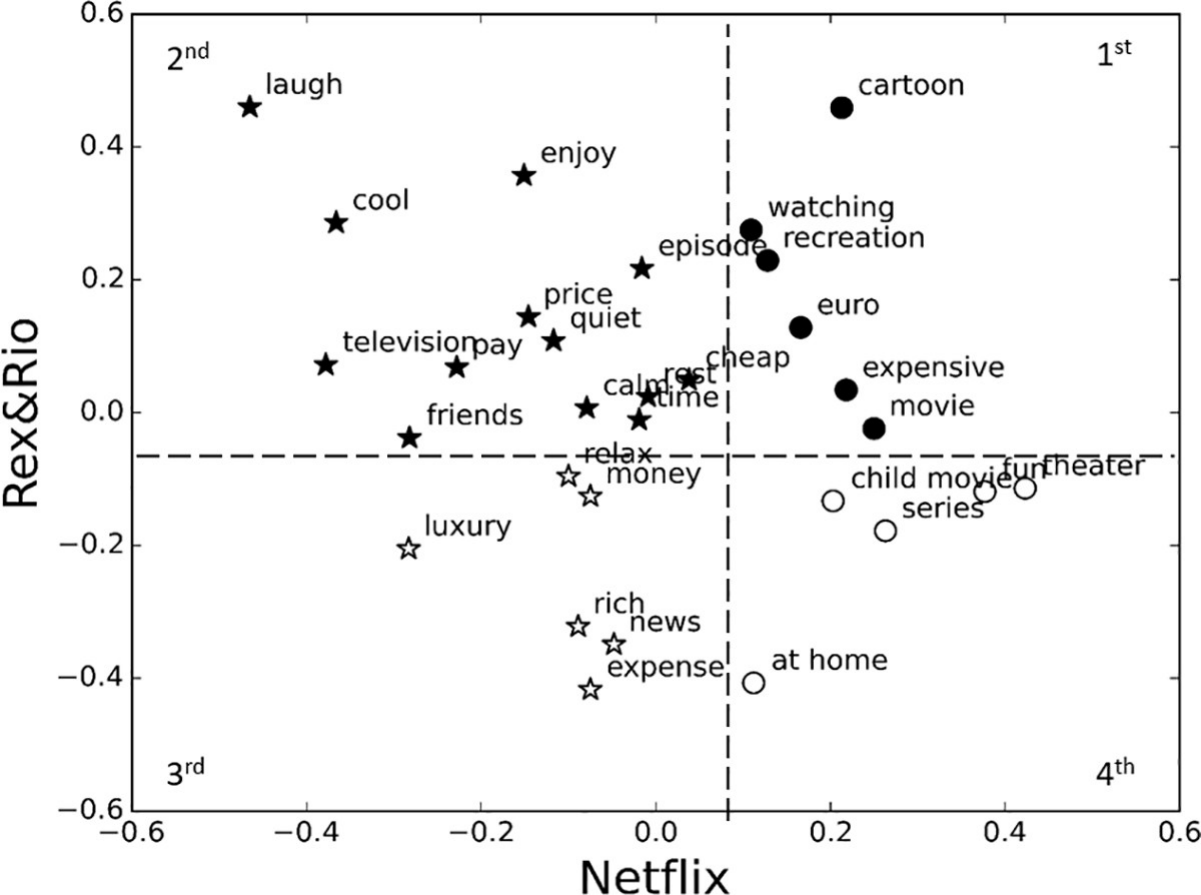
Cluster intersection of the two companies *Netflix* (horizontal axis) and *Rex&Rio* (vertical axis). Cluster Intersection between the two companies. Black and white colors represent Cluster 1 and 2 for *Rex&Rio*, and stars and circles Cluster 1 and 2 for *Netflix* (see *Single-brand analysis*). Recall that Cluster 1 contains smaller (or more positive) N400 responses and, hence, is more associated with the brand. In contrast, Cluster 2 is composed by larger (thus more negative) N400 responses and, therefore, is considered less related with the brand.

We also evaluated the associations that are shared or not shared between the 2 brands. Recalling the cluster solution of Analyses 1 and 2, we now focus on their intersections. We found an interesting correspondence when intersecting the previously defined clusters. Indeed, the intersection naturally defines 4 quadrants (Fig 6): the 1^st^ quadrant contains the intersection of Cluster 1 of both companies (related associations), the 2^nd^ quadrant represents the intersection between Cluster 1 of *Rex&Rio* and Cluster 2 of *Netflix*, the 3^rd^ quadrant the intersection between Cluster 2 of both companies (unrelated associations), and the 4^th^ quadrant the intersection between Cluster 2 of *Rex&Rio* and Cluster 1 of *Netflix*. In other words, while the 2^nd^ (related to *Rex&Rio* but unrelated to *Netflix*) and 4^th^ (related to *Netflix* but unrelated to *Rex&Rio*) quadrants contain brand associations not shared by the brands, the 1^st^ (related to both *Netflix* and *Rex&Rio*) and 3^rd^ (unrelated to both *Netflix* and *Rex&Rio*) quadrants include associations shared by the brands. For instance, we observe that in the 2^nd^ quadrant we mostly have the *Relaxation*-related words (e.g. laugh, enjoy, cool, quiet, calm), whereas in the 4^th^ quadrant mostly the *Television*-related words (e.g. child movie, theater, series, at home).

**Fig 7:**
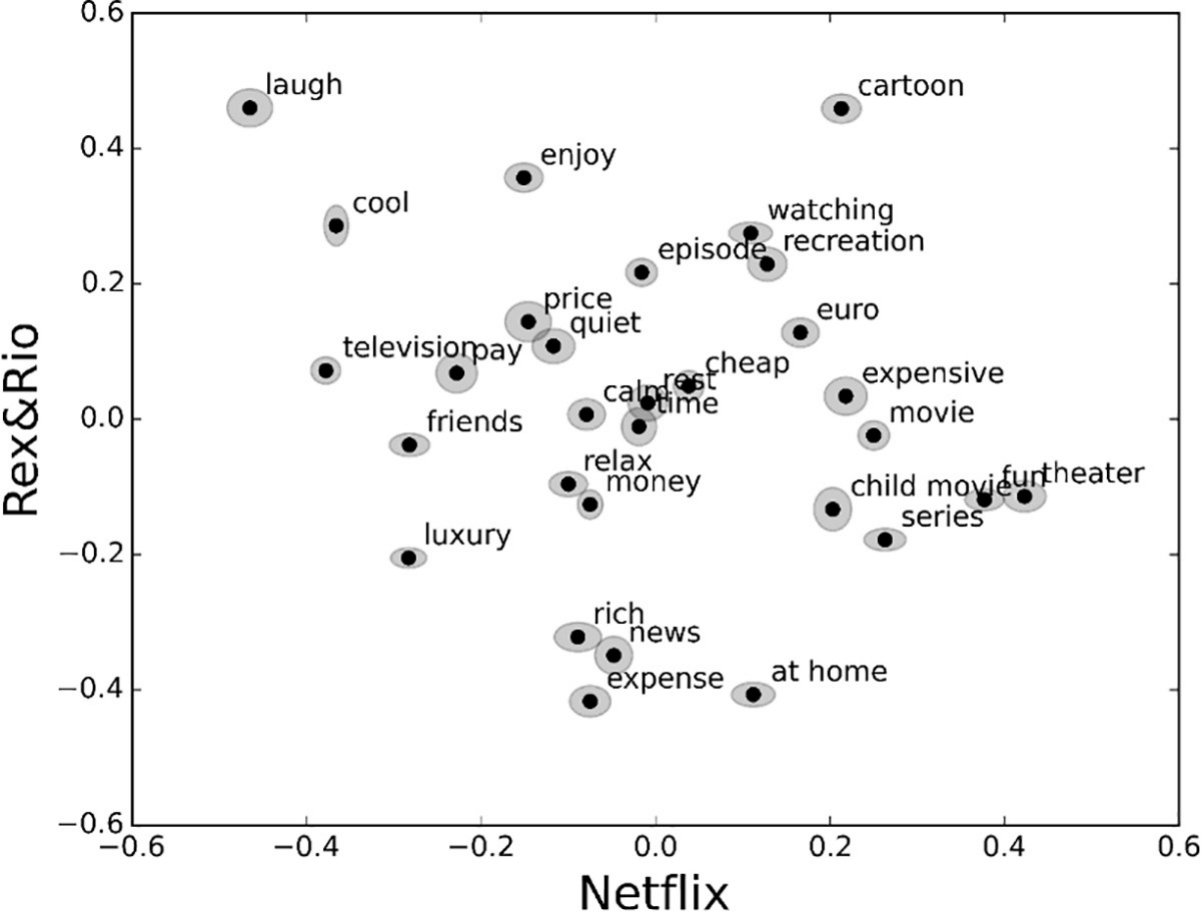
Stability assessment. Dots represent mean N400 response strengths for *Netflix* (horizontal axis) and *Rex&Rio* (vertical axis); ellipses represent standard deviations (horizontal and vertical ellipse axes are for *Netflix* and *Rex&Rio*, respectively). Means and standard deviations calculated by leaving one subject out.

To assess the stability of single-word N400 response strengths (see *Proposed single word calculation*) across subjects we plotted in *Rex&Rio* vs. *Netflix* space the means of the N400 responses and their standard deviations (as ellipses) calculated by leaving one subject out. We observe that, as shown in Fig 7, the ellipses are quite small, implying that the single word responses are stable.

## Discussion

In theories on consumer behavior, a brand is defined as a mental construct used by customers to distinguish products of one brand from another based on its attributes [58]-[60]. Therefore, the company is responsible for the identity of its brands by creating sufficiently differentiated products. But how a brand is actually perceived depends on a set of associations and beliefs about the brand (brand associations). Strong brand associations can support the consumer to consider and purchase products when retrieval stimuli are presented in commercial messages [61].

The theory of semantic network and spreading activation is rooted in neurolinguistics where the N400 ERP component is well-documented in connection to semantic priming [46],[62]-[65]. The N400 has been used as a measure of the association strength between two more- or less related concepts [55]. For instance, when the prime is the name of an animal and the target a non-living object then the N400 amplitude evoked by the target is larger compared to the case where the prime and target are both names of animals.

Some studies considered N400 as a tool for analyzing brand extensions based on brand associations. For example, it was found that the N400 correlates with the processing of brand categorization [35]: a product name can elicit N400 responses when primed with an untypical brand name (out-of-category). However, the authors did not provide any indication concerning the strengths of single those brand associations neither did they compare the brands.

In this study, we aimed to evaluate the alignment between brand image and brand identity. Keller defined brand image as "perceptions about a brand as reflected by the brand associations held in consumer memory" [66]. We introduced a new tool based on N400 measurements for analyzing single brand associations (brand image) and how well they correspond to the brand’s identity. After showing the video ads of the 2 brands, we gauged mental associations with 2 brand logos using N400 responses as a proxy. We performed 3 analyses: first the 2 brands were independently analyzed in order to identify weak and strong brand associations (Analysis 1 and 2), then we looked for the brand associations that were shared (common associations) and not shared (distinctive associations) between the brands (Analysis 3). We adopted a data-driven approach (clustering) to identify natural groups of associations as they are likely to reside in our participants’ minds.

In van Vliet et al.’s work [47] it was shown that the N400 can be used for identifying natural clusters. However, in their study, the stimuli were taken from two clearly separated categories (animals vs. furniture), with an equal number of stimuli for both categories, against which the found clusters could be checked. On a more technical note, the target word responses were simply averaged across subjects and clustering performed by block modeling assuming 2 equally-sized clusters (i.e., an equal number of data points).

In contrast, we did not rely on any of those assumptions. In fact, we defined a new measure (stability index) to determine the optimal number of clusters. As a result of our clustering analysis (Analysis 1 and 2), we identified 2 clusters for both brands: Cluster 1 with the most positive N400 responses and Cluster 2 with the most negative ones.

When comparing these clusters to the 3 original categories, we observed that for *Netflix* (Analysis 1) our participants seemed to associate the brand with *Television*, as Cluster 1 was composed mostly by words from this category (7 out of 11), but not with *Relaxation* and *Price* as 80% of the words from the 2 categories were in Cluster 2. In contrast, for *Rex&Rio* (Analysis 2), a correspondence with *Relaxation* could be observed as 80% of the words from this category were included in Cluster 1. We also analyzed the correlation between the N400 responses of the two brands (Analysis 3). When intersecting the obtained clusters, we confirmed that the unique association for *Netflix* was *Television* whereas for *Rex&Rio* it was *Relaxation*. This seems to suggest that the 2 brands are perceived differently, which in turn could provide new entry points for a marketing campaign. However, this difference is not visible in the behavioral data where the two brands exhibited the same outcome: strong association with *Television* and *Relaxation*, whereas weak association with *Price*. Heylen and co-workers [39] suggest that brand image consists of both explicit and implicit components. The latter they defined as "an emotional rather than a rational basis […] the implicit, internal features that are experienced by the primal, subconscious brain" while the former as "the explicit, external features that are observed by the rational". Many associations are also unconsciously stored in a nonverbal mode [67], hence, we conjecture that the mental evaluation of brands, here measured in terms of N400 responses, is not only subject to conscious processing and therefore cannot be fully grasped with the–possibly rationalized–explicit answers returned in questionnaires.

## Conclusion

The proposed EEG-based tool was applied to gauge consumers’ perceived associations to two video-on-demand brands by measuring their N400 responses to individual brand logo–word associations. A large N400 response is indicative of a low association strength in the customer’s mind and vice-versa. In this study, we used such responses to identify two word groups for each brand, with weak- and strong brand associations, and to single out the associations shared or not shared by the two brands. These findings could be used to the advantage of the (neuro)marketer when deciding on which marketing stimuli to use (or not to use) when aiming to differentiate the brand from its competitors or to narrow the gap between perceived and desired brand associations.

## Supporting information

S1 Appendix Cluster analysis. (DOCX)

S1 Fig N400 responses associated with single trials. Grids representing the normalized N400 values associated with single trials. White entries correspond to missing values (removed trials). Left panel: responses for Rex&Rio. Right panel: responses for Netflix.
(TIF)

S2 Fig Netflix analysis. Left panel: dendrogram representing the result of hierarchical clustering when including all subjects (*complete solution)*. Right panel: stability index as a function of number of clusters. The highest stability index was 0.9.
(TIF)

S3 Fig Rex&Rio analysis. Same conventions as in S2 Fig. The highest stability index was 0.72.
(TIF)

S1 Table Participants’ responses per category. “No answer” refers to trials with no button response, “HD” to trials where the EEG amplitude exceeded our 70μV threshold on any of the channels. Both types of trials were removed. *Participant with less than 30 trials (excluding Unrelated category), removed. In total 1767 out of 2080 trials were considered in the analysis.
(PDF)
