# Supplementary material for: Measuring brand association strength with EEG: A single-trial N400 ERP study
Source: PLoS One. 2019 Jun 10;14(6):e0217125. doi: 10.1371/journal.pone.0217125 (PMC6557491; doi:10.1371/journal.pone.0217125)
Supplement: S1 Appendix — (DOCX) [file pone.0217125.s001.docx]

Supplementary information

**S1 Fig. N400 responses associated with single trials.** Grids representing the normalized N400 values associated with single trials. White entries correspond to missing values (removed trials). Left panel: responses for Rex&Rio. Right panel: responses for Netflix.

**S1 Appendix. Cluster analysis**

We opted for an agglomerative, hierarchical clustering procedure as it does not rely on any prior assumption about the number of clusters. At each step, a pair of clusters is merged when optimal, given an objective function. In this case, the objective function is the error sum of squares, known as Ward's minimum variance (for a detailed description, see [1],[2]). The cluster analysis was performed in Python [3]. The result is a dendrogram, a tree-like structure that visualizes the sequence of merged clusters (see the left panel in S2 Fig for an example dendrogram).

***Number of clusters and stability analysis***

The optimal number of clusters can be obtained by ‘cutting’ the dendrogram at an appropriate level. To this end, we adopted and extended the similarity measure of Fowlkes and Mallows [4], which is based on a comparison of two or more hierarchical clustering outcomes on the same set of objects. For a given number of clusters (thus, a given level in the hierarchy), the method calculates a similarity measure, ranging between 0 and 1 with 1 corresponding to identical clustering outcomes.

On this basis, we developed our *stability analysis*: first, we applied the *Single-brand analysis* including all subjects (*complete solution*), then one subject was discarded and the *Single-brand analysis* reran with the remaining subjects (*leave-one-out solution*); second, the obtained *leave-one-out solution* was compared to the *complete solution* using Fowlkes and Mallows’ similarity measure; third, the last 2 steps were repeated each time leaving out another subject until all of them were considered; and, finally, the series of obtained similarity measures were averaged. The outcome of the *stability analysis* is, thus, a set of averaged similarity values, each one representing the similarity for a specific level in the dendrogram. We further call this value the *stability index*. The number of clusters corresponding to the highest stability index was taken as optimal. We refer to the right panel of S2 Fig for an example of the *stability index* for different numbers of clusters.

***Netflix* clustering analysis**

The outcome of the stability analysis applied on the *Netflix* data indicates the 2-cluster case as optimal with a stability index equal to 0.90. This high stability can be interpreted as a high consistency across subjects. The results are summarized in S2 Fig.

**S2 Fig. Netflix study.** Left panel: dendrogram representing the result of hierarchical clustering when including all subjects (*complete solution)*. Right panel: stability index as a function of number of clusters. The highest stability index was 0.9.

***Rex&Rio* clustering analysis**

We repeated the clustering analysis but now on the *Rex&Rio* data. As shown in (Fig. 6, right panel), it is clear that the best solution is again obtained for the 2-cluster case with a stability index of 0.72.

**S3 Fig. Rex&Rio study.** Same conventions as in S2 Fig. The highest stability index was 0.72.

**S1 Table. Participants’ responses per category.** “No answer” refers to trials with no button response, “HD” to trials where the EEG amplitude exceeded our 70µV threshold on any of the channels. Both types of trials were removed. *Participant with less than 30 trials (excluding Unrelated category), removed. In total 1767 out of 2080 trials were considered in the analysis.

**References**

1. Lance, Godfrey N., and William Thomas Williams. "A general theory of classificatory sorting strategies: 1. Hierarchical systems." *The computer journal* 9.4 (1967): 373-380.
2. Cormack, Richard M. "A review of classification." *Journal of the Royal Statistical Society: Series A (General)* 134.3 (1971): 321-353.
3. Van Petten, Cyma. "Examining the N400 semantic context effect item-by-item: Relationship to corpus-based measures of word co-occurrence." *International Journal of Psychophysiology* 94.3 (2014): 407-419.
4. Fowlkes, Edward B., and Colin L. Mallows. "A method for comparing two hierarchical clusterings." *Journal of the American statistical association* 78.383 (1983): 553-569.
