# Supplementary figures and images for: Measuring brand association strength with EEG: A single-trial N400 ERP study
Source: PLoS One. 2019 Jun 10;14(6):e0217125. doi: 10.1371/journal.pone.0217125 (PMC6557491; doi:10.1371/journal.pone.0217125)

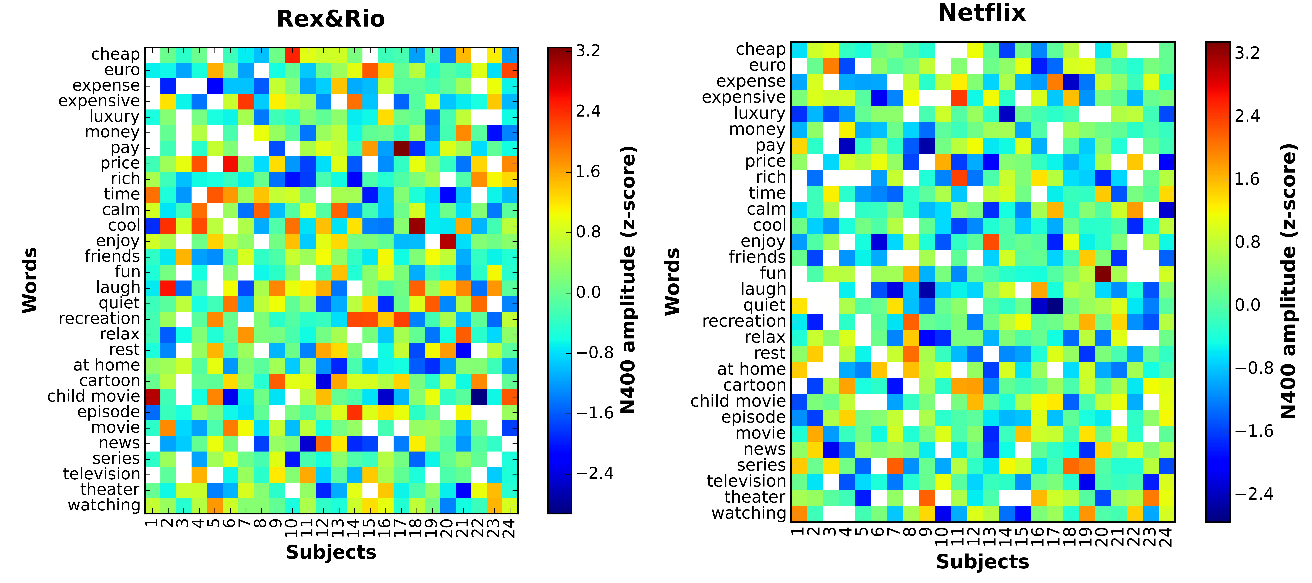

Supplement: S1 Fig — Grids representing the normalized N400 values associated with single trials. White entries correspond to missing values (removed trials). Left panel: responses for Rex&Rio. Right panel: responses for Netflix. (TIF) [file pone.0217125.s002.tif]

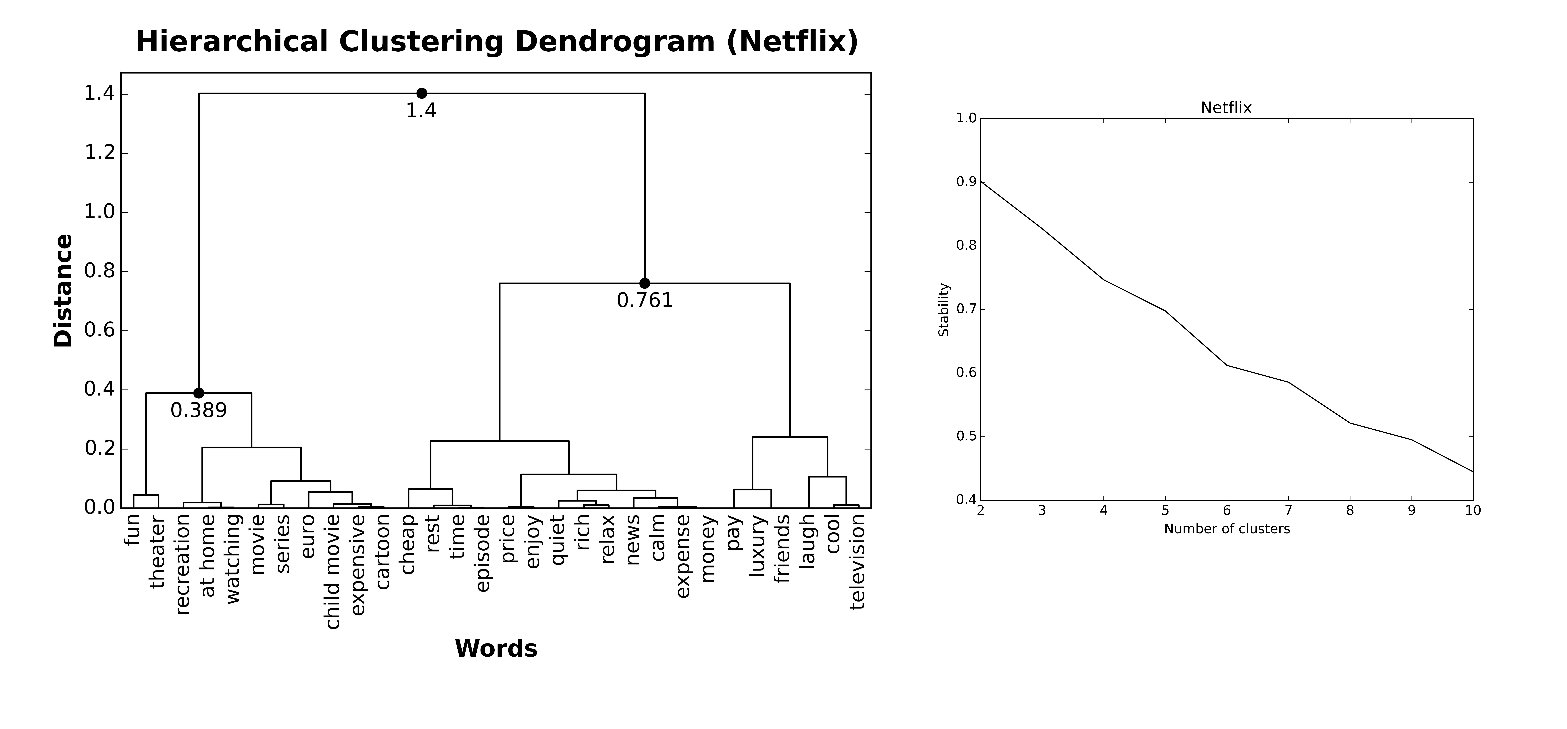

Supplement: S2 Fig — Left panel: dendrogram representing the result of hierarchical clustering when including all subjects (complete solution). Right panel: stability index as a function of number of clusters. The highest stability index was 0.9. (TIF) [file pone.0217125.s003.tif]

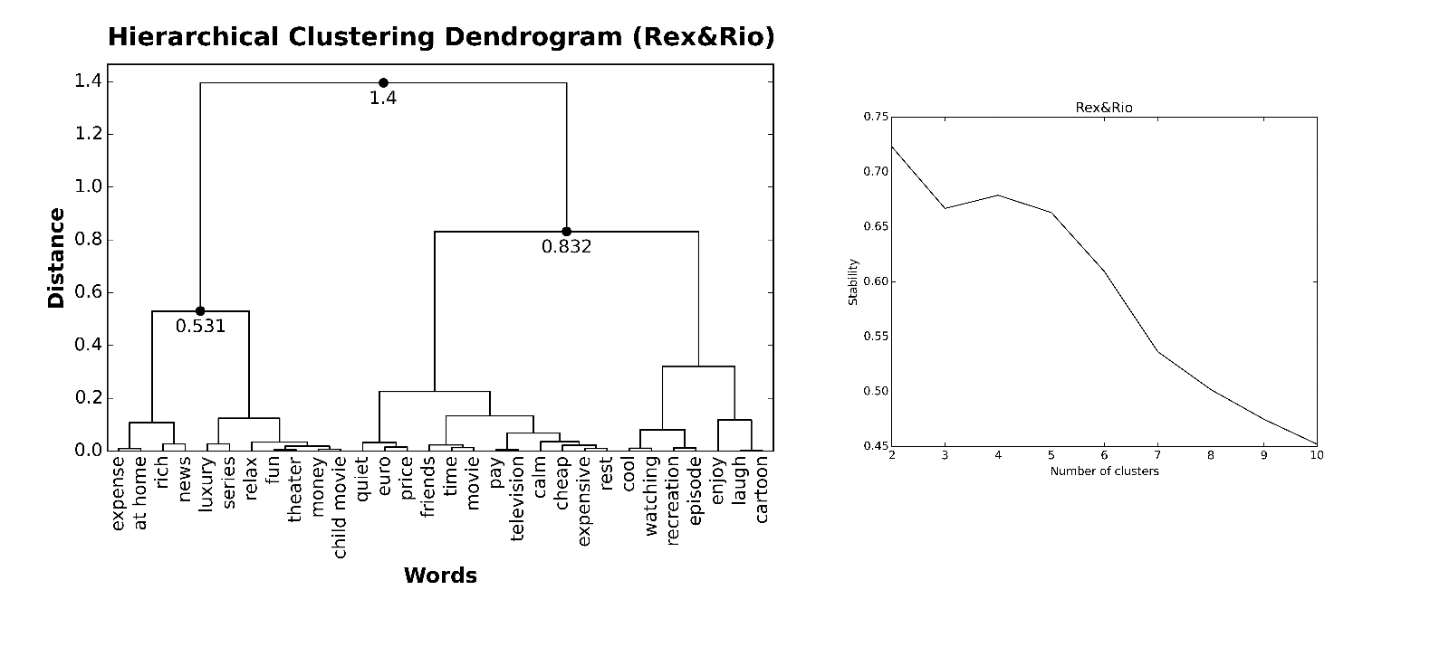

Supplement: S3 Fig — Same conventions as in S2 Fig. The highest stability index was 0.72. (TIF) [file pone.0217125.s004.tif]
