# Supplementary material for: Measuring brand association strength with EEG: A single-trial N400 ERP study
Source: PLoS One. 2019 Jun 10;14(6):e0217125. doi: 10.1371/journal.pone.0217125 (PMC6557491; doi:10.1371/journal.pone.0217125)
Supplement: S1 Table — “No answer” refers to trials with no button response, “HD” to trials where the EEG amplitude exceeded our 70μV threshold on any of the channels. Both types of trials were removed. *Participant with less than 30 trials (excluding Unrelated category), removed. In total 1767 out of 2080 trials were considered in the analysis. (PDF) [file pone.0217125.s005.pdf]

Participants	Trials removed	"no answer" /"HD"	Netflix-Price	Netflix-Relaxation	Netflix - Television	Netflix - Unrelated	Rex&Rio-Price	Rex&Rio - Relaxation	Rex&Rio - Television	Rex&Rio - Unrelated
1	17	4/13	7	8	9	9	5	10	8	7
2	5	2/3	9	8	9	9	10	10	10	10
3	27	1/26	6	5	7	9	6	6	6	8
4	8	2/6	9	8	9	10	8	10	10	8
5	19	6/14	8	6	9	7	6	7	9	9
6	6	3/3	10	9	7	9	10	9	10	10
7	13	1/12	9	9	9	7	8	8	9	8
8	12	2/11	8	8	7	9	8	8	10	10
9	4	2/2	8	10	10	9	10	10	9	10
10	15	3/13	7	9	7	9	9	10	8	6
11	4	3/1	9	10	9	10	10	9	10	9
12*	38	9/30	5	5	9	8	1	4	6	4
13	7	2/5	8	8	10	8	9	10	10	10
14	2	2/0	10	9	10	10	9	10	10	10
15	5	1/4	10	10	9	9	10	8	10	9
16	9	2/7	9	9	8	10	8	9	8	10
17	3	3/0	9	10	10	10	9	10	9	10
18	8	2/6	8	10	9	8	10	10	9	8
19	1	1/0	10	10	10	9	10	10	10	10
20	4	3/1	8	10	10	10	10	9	9	10
21	3	0/3	9	10	10	10	9	10	9	10
22	5	4/1	9	10	9	8	10	10	10	9
23	19	2/17	7	6	10	8	6	9	8	7
24	18	14/5	8	6	8	7	8	9	6	10
25	2	0/2	10	10	10	9	10	10	9	10
26*	59	55/14	4	2	3	0	5	1	3	3
**Tot**	**313**	**129/199**	**214**	**215**	**227**	**221**	**214**	**226**	**225**	**225**
